# A Comparative Assessment of Three Mandibular Retention Protocols: A Prospective Cohort Study

**DOI:** 10.3290/j.ohpd.b2805357

**Published:** 2022-03-14

**Authors:** Jan Christian Danz, Isabella Scherer-Zehnder, Nikolaos Pandis

**Affiliations:** a Lecturer, Department of Orthodontics and Dentofacial Orthopedics, University of Bern, Bern, Switzerland, Designed and planned the study, treated the patients, wrote the manuscript.; b Postgraduate Student, Department of Orthodontics and Pediatric Dentistry, University of Zürich, Zürich, Switzerland. Co-first author, recruited the patients, took and recorded the measurements, wrote the manuscript.; c Associate Professor, Department of Orthodontics and Dentofacial Orthopedics, University of Bern, Bern, Switzerland. Statistical analysis, revised the manuscript.

**Keywords:** adverse effects, bond failure, enamel adhesion, fixed retainers, relapse, retainer failure, retention, stability of orthodontic treatment

## Abstract

**Purpose:**

Fixed retainers have been advocated for the prevention of anterior mandibular crowding after orthodontic treatment. However, limited data is available to help clinicians choose a retention protocol that is acceptable in terms of stability, emergencies, and side effects in the long term. It was the aim of this study to assess survival and alignment stability of the 0.016” x 0.022” stainless steel wire compared to more common protocols.

**Materials and Methods:**

Three different mandibular fixed retention protocols were compared in 600 consecutive patients: 1. 0.0215” multistrand wire (MW) with separate curing of resin and composite; 2. 0.016” x 0.022” stainless-steel wire with simultaneous curing of resin and composite (SS1C); and 3. 0.016” x 0.022” stainless-steel wire with separate curing of resin and composite (SS2C). The hazard rate for detachment across wire groups was assessed with a Cox frailty model.

**Results:**

Incisor alignment was maintained with all retention wires. One incisor with unexpected torque change was observed in group MW. The average annual emergency rate was below 2% for all three protocols. Fewer emergency visits were found in patients with solid steel wires than with multistrand wires. Detachment of the wire is the most common cause of emergency visits with no difference between wire types. Multistrand wires were more often damaged than were solid steel wires. There was no evidence that direct application of the composite on the uncured primer influenced retainer adhesion to the enamel.

**Conclusions:**

The mandibular anterior teeth can be predictably stabilised with a 0.016” x 0.022” stainless steel wire.

Stability of orthodontic treatment is fundamental for long-term performance and patient satisfaction.^5,20^ Because stability with removable retainers depends heavily on the cooperation of the patient,^1,3,4,6,9,13,23,31,35^ fixed retainers have become an important alternative to removable retainers, with evidence of compatibility with the periodontium.^1,2,8,14,18,21,28,33,35,36^

For fixed retention, round and rectangular stainless steel, β-titanium and fiberglass-reinforced wires have been proposed, with multistrand stainless steel wires being the most commonly used.^8,14,15,23,24,26,32,34,36^ A plethora of retention protocols have been reported, with variations in terms of the number of teeth attached (canines only or six anterior teeth), wire shape (straight vs looped), and bonding materials and methods.^8,24^

Adhesive failure was the most frequent fixed-retention complication, whereas loss of adhesion was less frequent among experienced practitioners.^11,13,16,24,29,32^ Failure to preserve alignment has been reported in connection with adhesive failure or with retainers bonded to canines only,^10,31^ with no difference in the risk of failure between indirect and direct bonding of multistrand wires.^7^

Unexpected post-treatment changes, such as movements due to torque changes between two adjacent incisors with flexible spiral wire retainers bonded on all six mandibular anterior teeth,^19,30,37^ can lead to compromised periodontal structures and tooth loss.^12^ To overcome these problems, Katsaros et al^9^ proposed the use of sandblasted 0.016” x 0.022” stainless steel retainers bonded to all six sandblasted mandibular anterior teeth with the 0.022-inch-plane in contact with the tooth surface.

A retention protocol which reliably maintains alignment, has no side effects, and requires minimum maintenance would be optimal for patients and practitioners.^26^ Because evidence on the efficacy of solid 0.016” x 0.022” stainless-steel retainers bonded to all six mandibular front teeth is not available, the aim of this study was to evaluate the success, maintenance, and side effects of: 1. sandblasted 0.016” x 0.022” stainless-steel wires with simultaneous curing of resin and composite (SS1C) and 2. plain 0.016” x 0.022” stainless-steel wires with separate curing of resin and composite (SS2C), and compare them to a control group (MW) in which 0.0215” multistrand wires were bonded using direct adaptation of the wire and simultaneous curing of resin and composite, in which unexpected post-treatment changes have been previously reported.^12,19,30^

## Materials and Methods

### Study Design

A total of 600 consecutive patients were selected and their data were recorded from patient files. Digital casts were made by one of the authors (I.Z.). The only inclusion criterion was placement of a mandibular canine-to-canine retainer by one operator, and the only exclusion criterion was one or more missing mandibular front teeth.

### Interventions

All patients had been previously treated with self-ligating appliances (SPEED System). In the control group MW, 0.0215” multistrand wires (Penta One, Masel; Carlsbad, CA, USA) were directly adapted and bonded with separate light curing of primer and composite. In the group SS1C, 0.016” x 0.022” stainless steel wires (permachrome resilient straight lengths, 3M Oral Care; St Paul, MN, USA) were prepared on plaster models and bonded using a silicone transfer guide by simultaneous light curing of the primer (Transbond XT Primer, 3M Oral Care) and the composite (Transbond LR, 3M Oral Care). In the group SS2C, 0.016” x 0.022” stainless steel wires (304V Vacuum Arc Remelted stainless-steel straight lengths, Highland Metals; Franklin, IN, USA) were prepared on models and bonded with separate light curing of the primer and the composite. All wires were adapted to the arch form and in contact with every lingual tooth surface. Rectangular bends at wire ends within the composite were added to prevent shifting of the wire within the composite.

All patients were examined one week before retainer bonding, and any calculus on the mandibular front teeth was removed. Patients were advised to rinse twice daily with 0.2% chlorhexidine for 30 s. The retainer was inserted before appliance removal using the following protocols: After the lingual surfaces were thoroughly scaled and polished, the entire tooth surfaces were etched for 30 s with 35%–37% phosphoric acid. The etching gel was rinsed for approx. 10 s per tooth and the completeness of the etching pattern was checked by drying the lingual surfaces. After the application and thinning of the primer coating with air, the primer was polymerised for 12 s per tooth (only in the control group and in group SS2C where primer and composite were cured separately). The retainer was cleaned with 70% alcohol, dried, inserted and held in position with the left thumb. Composite was added and cured for 12 s, first onto the lingual surface of the right mandibular canine, followed by the left canine and the incisors. The silicone transfer guide was removed after light curing of the canines (only for SS1C). The composite was smoothed with a microbrush (thixotropy) and excess was removed with a probe to achieve a thickness of 0.5 mm over the wire. Primer and composite margins were checked, and any excess was removed with a scaler or a carbide bur. All patients received retainer instructions on how to clean the retainer with a single brush.

All patients were advised to have regular check-ups after one month and then at least every three months for at least one year after retainer insertion. Patients with growth disturbances were observed for longer periods of time. Adhesive or wire failures were recorded and repaired immediately. In the presence of defective wires or unexpected post-treatment changes, the patient was given the options of retreatment and placement of a new wire or the removal of the defective wire.

Blinding of the practitioner was not possible because of the different wire materials.

This study adhered to the Principles of the Declaration of Helsinki, and ethical approval was given from the Swiss Ethics Committee on research involving humans 2019-00264.

### Irregularity Index

**Fig 1:**
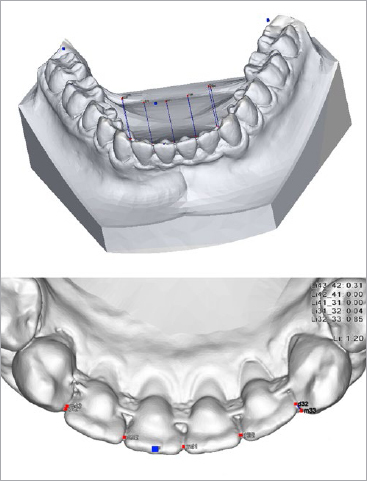
Little’s irregularity index in the mandibular front (Iil) was digitally constructed. First, the occlusal plane was set as a plane through the second buccal cusp of both first mandibular molars and the incisal edge of the most proclined lower incisor. Then, the anatomic contact points of each mandibular incisor were projected perpendicularly on the occlusal plane and the five linear displacements to the projected anatomic contact point of the adjacent tooth were summed.

At the end of treatment, plaster models were digitised using an Ortho Insight 3D Desktop Scanner (Motionview; Chattanooga, TN, USA). The mandibular arch was imported in the Archimedes Geo3D application (raumgeometrie.de, Göttingen, Germany) and geometric designs corresponding to Little’s analogous index^25^ were constructed for each model (Fig 1). Little’s irregularity index reflects the sum of the distances between the vertical projections on the occlusal plane of the five pairs of contact points mesial to the mandibular canines.^25^

### Outcomes

The primary outcomes were success (no increase in clinically relevant contact point displacements >0.5 mm) and absence of unexpected post-treatment changes. The secondary outcomes were emergency visit, detachment, and wire failure. An “emergency visit” was defined as an appointment for repair of either adhesive and/or retainer wire failure. Detachment from a tooth was defined as a fracture of the composite exposing the wire, or a loosening of the composite from the tooth so that the wire had to be reattached. Wire failure was defined as a defect of the wire which required replacement. The emergency rate was calculated by dividing the number of emergencies by the patient’s total follow-up time. The detachment rate per tooth was calculated by dividing the number of detachments by the patient’s follow-up time and by 6 (bonded teeth).

### Statistical Analysis

The number of emergency visits, detachments and wire failures across wire groups were compared using Fisher’s exact test. The agreement of double measurement error was assessed using the intracluster correlation coefficient (ICC). The hazard ratio for detachment across wire groups was calculated with a Cox frailty model.

## Results

The median age of patients was 14.7 (range 11.1 to 57.0) years at retainer placement, and the median follow-up period was one year. The median treatment time was 497 days and resulted in a median Little’s index of 0.32 mm. Descriptive statistics of the studied population are shown in Table 1. The ICC for agreement of double measurements for Little’s irregularity index was 0.93 (95% CI: 0.89, 0.98).

**Table 1:** Characteristics of all patients

	Minimum	25^th^ percentile	Median	75^th^ percentile	Maximum
Age	11.1	13.6	14.7	16.0	57.0
Follow-up time	0	284	364	474	2093
Treatment time	76	380	497	618	1900
Little’s index	0.00	0.19	0.32	0.51	1.95

**Fig 2:**
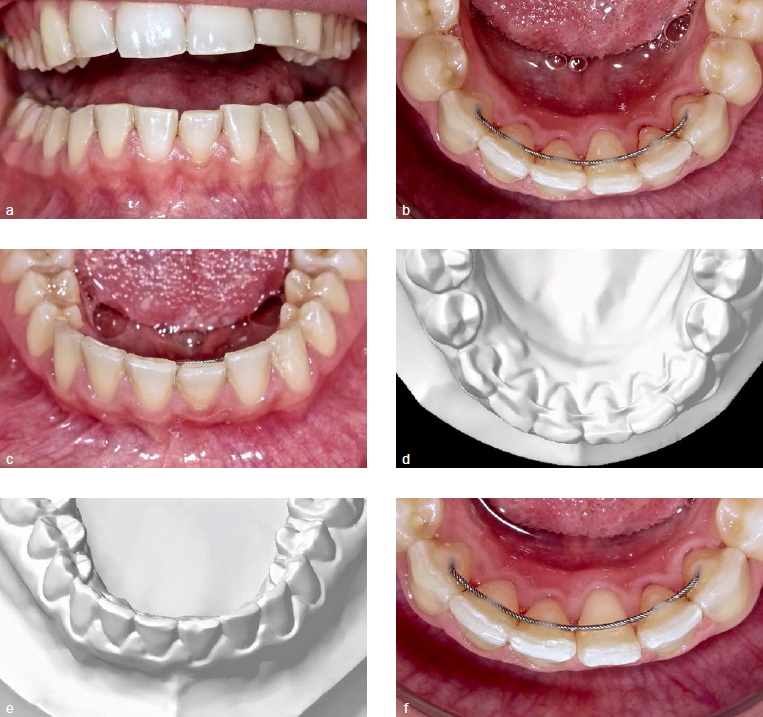
Digital models at debonding and intraoral images four years after show the development in the patient with a multistranded wire. An unexpected torque movement of the mandibular left first incisor is evident with intact composite and no history of repair (a, b and c). Mandibular incisor 31 had an enamel fracture and a rotation distally outward, but no relevant torque differences at debonding (d and e). There was no spontaneous correction one month after removal of the composite (f). The patient refused re-treatment to adjust torque of 31 before retainer renewal, and the retainer was removed to prevent additional unexpected torque movements.

The incisors were stabilised successfully in all patients but one from the MW group, whose alignment deteriorated due to unexpected post-treatment changes (Fig 2). Unexpected torque changes were not observed in the groups SS1C and SS2C.

The secondary outcomes are listed in Table 2. Multistrand wires caused more emergencies per year than did solid steel retainers (p < 0.01), and the number of emergency visits did not differ between SS1C and SS2C (p = 0.85).

**Table 2:** Main outcomes

Main outcomes	MW	SS1C	SS2C
n	200	200	200
Stable	199	200	200
Adhesive failure	58	28	19
No. of patients with detachments	31	20	13
Retainer wire failure	6	0	1
Emergencies	50	23	17
Average Little’s index	0.90	0.45	0.40
	MW	SS1C	SS2C
Annual emergency rate per patient (average)	1.36%	1.26%	0.74%
Annual detachment rate per tooth (average)	2.92%	2.37%	1.37%
	MW	SS	
Annual wire failure rate (average)	1.160%	0.195%	

Rates were calculated by dividing the number of events by the patient’s total follow-up time.

**Fig 3:**
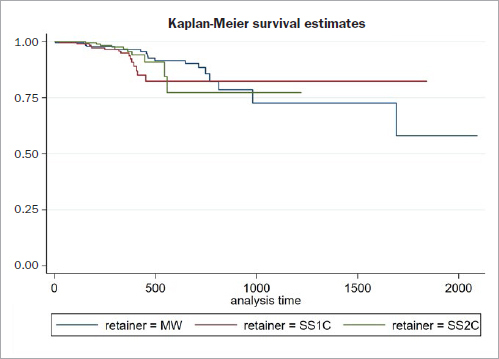
The hazard of detachment was calculated using a Cox frailty model. The protocols with 0.0215” multistranded wires (MW) and separate curing of resin and composite, with sandblasted 0.016” x 0.022” stainless steel wires and simultaneous curing of resin and composite (SS1C) and with 0.016” x 0.022” stainless steel wire and separate curing of resin/composite (SS2C) showed similar survival estimates of attachment.

**Fig 4:**
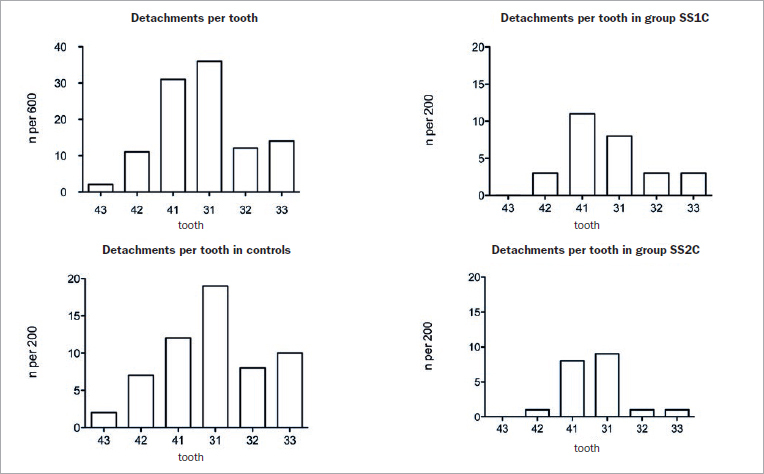
The central incisors were most prone to loosening of the wire, followed by the lateral incisors and the mandibular left canine. The mandibular right canine was least likely to have detached composite or composite fractures.

Detachments were the most common reason for an emergency treatment. Thirty-seven patients needed a wire reattached once, 13 patients twice, five patients three times, two patients five times and one patient nine times. The hazard ratio for detachments was higher for SS1C (1.58 CI 0.78 to 3.20) and SS2C (1.14 CI 0.51 to 2.57) compared to MW, but not statistically significant. The Cox frailty model showed no evidence of association between retainer type and detachments (Fig 3). Not all teeth were equally affected by detachments (Fig 4). The central incisors were most frequently affected by loosening of the bond or composite fractures.

Six out of 200 multistrand wires and one out of 400 solid steel wires failed (p < 0.01).

## Discussion

The alignment of the mandibular incisors was predictably ensured with fixed retention wires, as observed in numerous other studies.^6,10,13,22^ Solid steel wires are preferable to multistrand wires, as fewer wire failures and no unexpected torque changes were observed.

In an earlier study,^21^ round TMA wire bonded only to the mandibular canines proved to be free from unwanted displacement or root torque, whereas two displacements were reported in 135 multistrand retainers. Our study is consistent with these findings: solid steel wires were also free from unexpected post-treatment changes in contrast to multistrand wires, where torque changes were found. If unwanted torque movements of multistrand wires are detected, the wire should be removed immediately at least at the misaligned teeth. During an observation period of up to one month, a spontaneous correction can occur, and this follow-up period can be used to evaluate the options: retainer removal, retainer replacement after spontaneous correction, or re-treatment and retainer renewal.

Detachments of the wire were either at the composite-tooth interface or fractures of parts of the composite. The Cox frailty model revealed no statistically significant differences in detachments between wire types. The material properties of the composite were sufficient to withstand stress peaks resulting from high stiffness of solid steel wires when single teeth were loaded in vivo.

Another advantage of a solid wire is that detachment on one tooth can usually be repaired immediately before significant displacement has occurred, and without the risk of damaging the wire during composite removal and cleaning. To maintain incisor alignment in the long term, it is important to advise the patient to immediately contact the family dentist when a detachment is noticed, before any possible tooth displacement occurs.

Little’s irregularity index and its necessary measurements have by definition no values below zero. Proportional bias found in double measurements of Little’s index indicate better agreement for small values. Larger displacement of contact points seem therefore more difficult to measure than very small irregularities close to perfect alignment (0 irregularity). This can be seen as an advantage for summative measurements, as the agreement improves towards 0.

The higher detachment rates of 12%–50% per year reported in other studies^11,16,32^ may be attributable to bonding protocol, materials, moisture control, experience and training of the operator, lack of calculus removal one week before retainer insertion, lack of 0.2% chlorhexidine the week prior to retainer insertion, and presence of aprismatic enamel.^17^

Sandblasting of solid steel wires was recommended to enhance bond strength between wire and composite.^27^ The hazard ratio for detachment of the sandblasted wires (SS2C) did not differ significantly from other groups and was clinically similar to group SS1C. Nevertheless, sandblasting could prevent displacement of the wire in the composite. This was prevented in the methods described here by bending the ends.

Silicone transfer guides can be placed most precisely when the bond has not been cured (SS1C). The direct application of the composite onto the liquid primer had no negative clinical influence on the success of adhesion. Thus, it is not necessary to cure the bonding agent when using the transfer guide. When placing the wire manually (SS2C), the bonding agent should be cured beforehand. A negative influence of the silicone transfer guide on adhesion is unlikely, since in all three methods, the central mandibular incisors were mostly, but equally, affected by detachment.

The importance of moisture control, gingivitis and the amount of gingival crevicular fluid is supported by the finding that the first-bonded tooth (43) showed fewest failures. Factors such as size of the lingual surface, access to the tooth and bonding sequence may also contribute to the varying detachment pattern. Most detachments were found on the central mandibular incisors, indicating that in addition to the sequence and speed of bonding procedures, also the distance to the sublingual caruncles may be of importance.

## Conclusions

Stabilization of all six mandibular front teeth by a 0.016” x 0.022” stainless steel wire is predictable and generates minimum emergency appointments. Unexpected torque movements were not found in the solid steel wire groups. Detachment of the wire is the most common reason for an emergency visit, and can be repaired by renewal of the bonding site. Damage of a solid steel wire was rare.
